# Open questions - in brief: Beyond -omics, missing motor proteins, and getting from molecules to organisms

**DOI:** 10.1186/1741-7007-11-8

**Published:** 2013-01-31

**Authors:** Stephen J Benkovic, Julie Theriot, Dagmar Ringe

**Affiliations:** 1 Department of Chemistry, Penn State University, 414 Wartik Laboratory, University Park, PA 16802, USA; 2 Department of Biochemistry, Stanford University, Stanford, CA 94305, USA; 3 Rosenstiel Basic Medical Science Research Center, 654, Brandeis University, Waltham, MA 02454-9110, USA

## A word from the editorial team

To celebrate the tenth anniversary of *BMC Biology*, we asked our Editorial Board members to say what they thought were some important or interesting open questions in biology [1]. Contributions range from a paragraph or two, like the collection below, to slightly longer contributions, the first two of which are also published this month [2,3]. We will publish these short contributions regularly during the year, to reflect the interests and preoccupations of our Editorial Board and perhaps stimulate answers from our readers.

## How do we find the functional significance of -omic-scale protein interactions?

### Stephen Benkovic

There has been a recent proliferation of publications that describe the identification of intracellular cytosol-based multi-protein complexes in various organisms that can act as functional modules for diverse biochemical activities. The method of choice is generally a form of affinity purification coupled with tandem mass spectrometry. A recent publication highlights the identification of 13,993 high-confidence physical interactions among 3,006 stably associated soluble human proteins resulting in 622 putative protein complexes [4]. To show that these complexes are functional, their participation in the cell cycle, and their regulation are challenges of paramount importance for future research activities.

## Do bacteria really not have cytoskeletal motor proteins?

### Julie Theriot

Are there cytoskeletal motor proteins in bacteria? Bacteria have many sophisticated force-generating machines, but so far nobody has found a homolog or analog of the cytoskeletal linear stepper motors (myosin, kinesin, or dynein) even though bacteria do have homologs of these motors' respective cytoskeletal filament tracks (actin and tubulin). Myosin and kinesin represent a particular sub-group of the P-loop NTPases, a sub-group that is most closely allied with the Ras superfamily proteins, which also includes the Rho family responsible for cytoskeletal regulation, the Rab family responsible for identifying distinct organelles, and the Arf family necessary for targeted membrane fusion, all of which are eukaryotic cell behaviors. Is this entire group of proteins simply absent from bacterial genomes? If we gave some of these proteins to bacteria, what would they do with them?

## How can we get from molecules to organisms?

### Dagmar Ringe

When I studied biology all of it was phenomenological. We learned how organisms are constructed, how that construction changes with phyla, and how that information can be used to develop ideas about evolution. No one was thinking about how those processes are driven at the molecular level. Now we are still asking about evolution, but the focus has changed to the prediction of organism organization, how different parts relate to each other, and how the molecular level morphs into the organism level. In addition, questions are being asked how the molecular level can drive these processes.

The problem has become more complex than when I studied it. We want to understand how processes that span many orders of magnitude in scale and time are interrelated. Any one experiment can only probe a limited spatial and time frame. But, in order to obtain a complete picture, many such frames have to be integrated and combined. Such integration can only be accomplished by computational modeling that is becoming an integral part of the effort. A number of researchers are now combining theoretical approaches that link molecular structure to organismal-scale behavior, opening the possibility to deconstruct evolutionary origins of cellular organization.
